# Convalescent plasma and all-cause mortality of COVID-19 patients: systematic review and meta-analysis

**DOI:** 10.1038/s41598-023-40009-8

**Published:** 2023-08-09

**Authors:** Nora Mihalek, Dragana Radovanović, Otto Barak, Petar Čolović, Markus Huber, Gabor Erdoes

**Affiliations:** 1 Faculty of Medicine, University of Novi Sad, Novi Sad, Serbia; 2 Department of Anaesthesiology, Intensive Therapy and Care, Oncology Institute of Vojvodina, Sremska Kamenica, Serbia; 3 Faculty of Philosophy, University of Novi Sad, Novi Sad, Serbia; 4 Department of Anaesthesiology and Pain Medicine, Inselspital, University Hospital Bern, University of Bern, Freiburgstrasse, 18, 3010 Bern, Switzerland

**Keywords:** Immunization, Outcomes research

## Abstract

Insight into the clinical potential of convalescent plasma in patients with coronavirus disease (COVID-19) is important given the severe clinical courses in unvaccinated and seronegative individuals. The aim of the study was to investigate whether there is a survival benefit of convalescent plasma therapy in COVID-19 patients. The authors independently assessed randomized controlled trials (RCTs) identified by the search strategy for inclusion, extracted data, and assessed risk of bias. The binary primary outcome was all-cause mortality. Risk ratio (RR) of the convalescent plasma treatment (vs. best standard care) and its associated standard error (effect size) were calculated. A random-effects model was employed to statistically pool the effect sizes of the selected studies. We included 19 RCTs with 17,021 patients. The random-effects model resulted in an estimated pooled RR of 0.94 (95% CI 0.81–1.08, p = 0.33), showing no statistical evidence of the benefit of convalescent plasma therapy on all-cause mortality. Convalescent plasma therapy was not found to be effective in reducing all-cause mortality in COVID-19 patients. Further studies are needed to determine in which patients convalescent plasma therapy may lead to a reduction in mortality.

## Introduction

According to the World Health Organization (WHO), more than 640 million cumulative cases of coronavirus disease (COVID-19) and 6.61 million COVID-related cumulative deaths have been registered worldwide since the outbreak of severe acute respiratory syndrome coronavirus 2 (SARS-CoV-2) in December 2019^1^. The clinical manifestations of the disease have covered a broad spectrum ranging from no symptoms to mild, severe, life-threatening or fatal disease. A meta-analysis including forty-five nonrandomized, retrospective observational studies up to the March 15, 2020 assessed intensive care unit (ICU) admission rates as high as 10.9% among patients with COVID-19 (probably alpha variant)^2^, however ICU admission rate and all-cause mortality rate in patients infected with newer virus variants can show different results, which are currently not well defined. There is evidence that patients with hypertension and diabetes type II are at higher risk of developing lethal complications^3^.

There are several modalities for the treatment of COVID-19-related deterioration of the health condition. These have had varying degrees of success, with the use of convalescent plasma producing contradictory results in recently published RCTs^4–7^. Convalescent plasma therapy is a form of passive immunization that takes plasma obtained from a patient who recently recovered from a disease and transfuses it into a patient who is currently ill from the same disease^8^. The sick person benefits from the antibodies produced by the recovered person. This modality is considered effective in the treatment of infectious diseases that are caused by viruses such as flaviviruses, influenza viruses A, Ebola virus, and respiratory betacoronaviruses^9^. Nevertheless, there is insufficient evidence regarding the clinical efficacy of convalescent plasma therapy in the treatment of COVID-19.

The exact mechanism of action of convalescent plasma is not yet well defined; however, it is supposed that viral neutralization, antibody-induced cellular cytotoxicity, complement activation, and phagocytosis can play a major role in enhancing recovery^10–12^. For the high percentage of the world’s population not actively immunized through vaccination, passive immunization with measures such as convalescent plasma therapy remains a potentially appropriate treatment option for patients who develop a severe clinical course.

This meta-analysis aimed to investigate the clinical efficacy of convalescent plasma in severely or critically ill COVID-19 patients in order to find out whether patients who receive convalescent plasma in addition to the best standard medical care (or placebo) have a lower all-cause mortality rate, and whether a shorter time from symptom onset to plasma transfusion leads to any clinical benefit. It is especially important to gain insight into the clinical potential of convalescent plasma therapy in the treatment of COVID-19 patients, because there is poor evidence regarding the efficacy of the current vaccines in treating the SARS-CoV-2 Omicron variant (B.1.1.529), or further variants that could appear in the future. Clinicians need to know whether plasma therapy could be an appropriate alternative (or rescue therapy) if vaccines fail to induce an immune response, or if patients are unvaccinated.

## Results

### Search strategy

**Figure 1:**
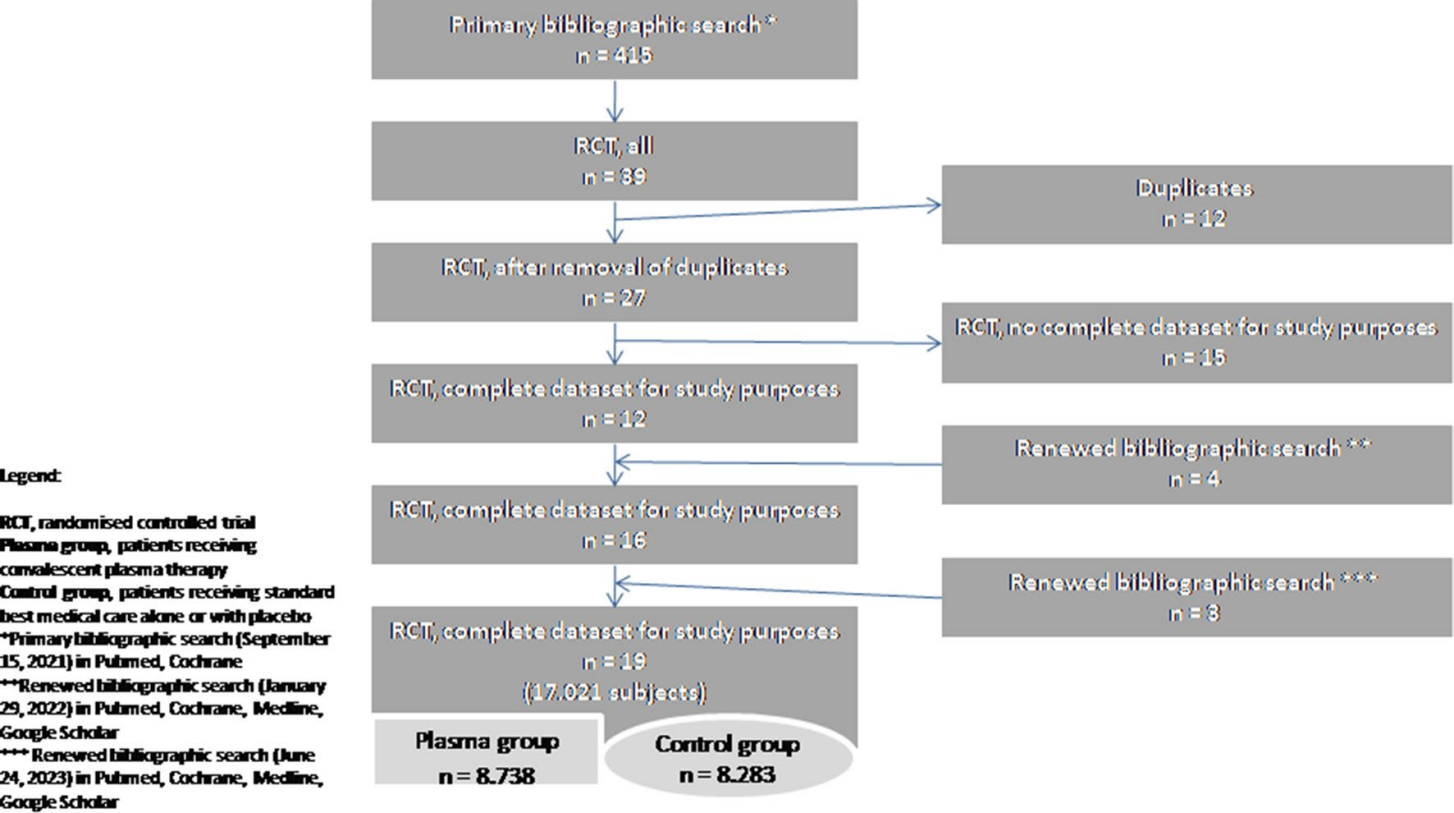
Study flow chart.

**Figure 2:**
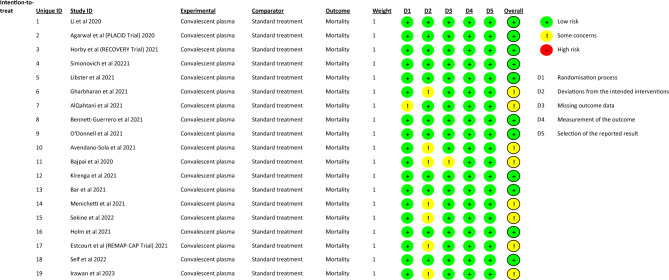
Risk of bias assessment according to the revised Cochrane risk-of-bias tool for randomized trials.

In an initial literature search on September 15, 2021 we found 39 RCTs in a total of 415 publications. Twelve articles were duplicates. After carefully screening the abstracts and full texts of the studies, 15 more publications were excluded because the studies did not provide information about relevant outcome data as e.g. all-cause mortality rate, co-morbidities or included patients enrolled in other clinical trials without focus on COVID-19 treatment. After the final screening, twelve randomized controlled trials^4–7,13–20^ were selected and included in our meta-analysis. After a renewed bibliographic search on January 29, 2022, using the same search strategy we identified four more RCTs^21–24^ which fulfilled the eligibility criteria, and as a result of an updated scan on June 24, 2023, we included three more studies in the analysis^25–27^. The total number of subjects included in the selected 19 RCTs was 17,021. Of those, 8738 participants received convalescent plasma in addition to the best standard care and 8283 subjects received the best standard care alone or with placebo, thus included in the control group (Fig. 1). The risk of bias assessment was performed using the revised Cochrane risk-of-bias tool for randomized trials^28^. We assessed the overall risk of bias as ‘low’ because sufficient information was reported and the method described was adequate in eleven studies, however in eight studies indicated ‘some concerns’, due to concerns regarding randomization and blinding techniques (Fig. 2).

### Pooled estimates

**Figure 3:**
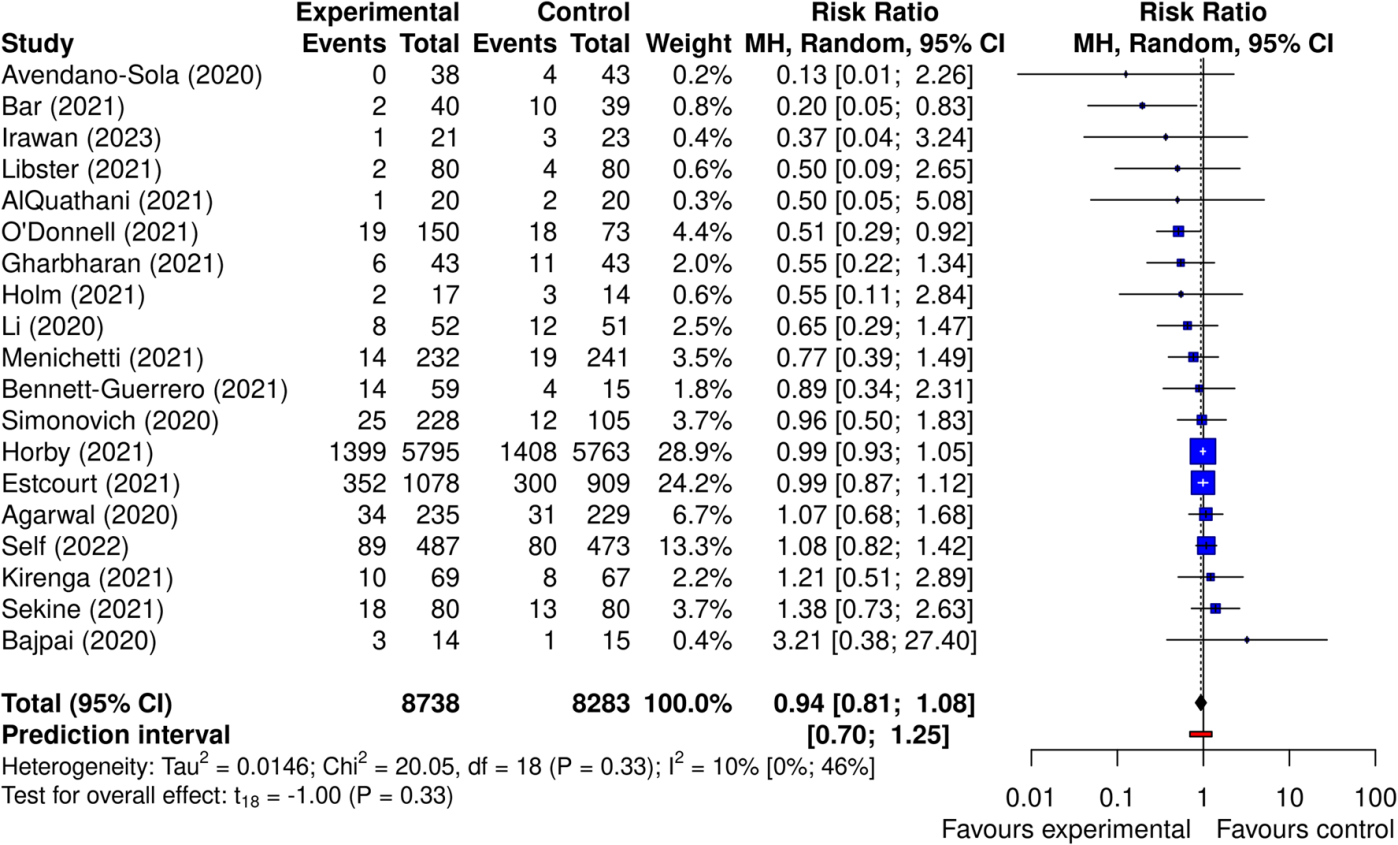
Forest plot comparing mortality between the two groups of patients. Forest plot comparing mortality in COVID-19 patients who received plasma therapy compared to the standard treatment based on a random effect statistical model. Effect size is the risk ratio (RR) and the statistical model features the Mantel–Haenszel method to pool the individual effect sizes, while the Paule-Mandel method is used to calculate the variance of the distribution of true effect sizes. The solid black diamond denotes the point estimate and its 95% confidence interval. The range of the prediction interval is depicted as a solid red line.

We started by examining the results of the random-effects model shown in the Forest Plot (Fig. 3). The random-effects model resulted in an estimated pooled risk ratio (RR) of 0.94 (95% CI 0.81–1.08, p = 0.33), thus showing no clear statistical evidence of the benefit of convalescent plasma therapy on all-cause mortality (outcome) in COVID-19 patients. The between-study heterogeneity variance was estimated at τ^2^ = 0.01 (95% CI 0.00–0.35) with an I2 statistic of 10.2% [95% CI 0.0–46.0%], indicating only low between-study heterogeneity. The prediction interval based on the random effects model ranges from RR = 0.70 to RR = 1.25, suggesting no clear preference for a particular treatment. Note that given the large sample sizes compared to other studies, the two RCTs by Horby^18^ and Estcourt^25^ contribute the most to the overall pooled effect, with a collective weight of 53.1%.

### Sensitivity analysis

Supplement 1 presents the inferred pooled risk ratios and associated p-values as well as the estimated variance of the distribution of true effect sizes as a function of the estimators of the between-study heterogeneity and the choice of Hartung-Knapp adjustment. While the mean estimate of the inferred pooled risk ratio can vary between 0.83 and 0.98 depending on the choice of estimator, the previous results of no statistical evidence of the benefit of convalescent plasma therapy on all-cause mortality (outcome) in COVID-19 patients is robustly supported by the sensitivity analyses (e.g. there is no estimator choice for which the pooled risk ratio is significantly different from 1).

### Influence analysis

**Figure 4:**
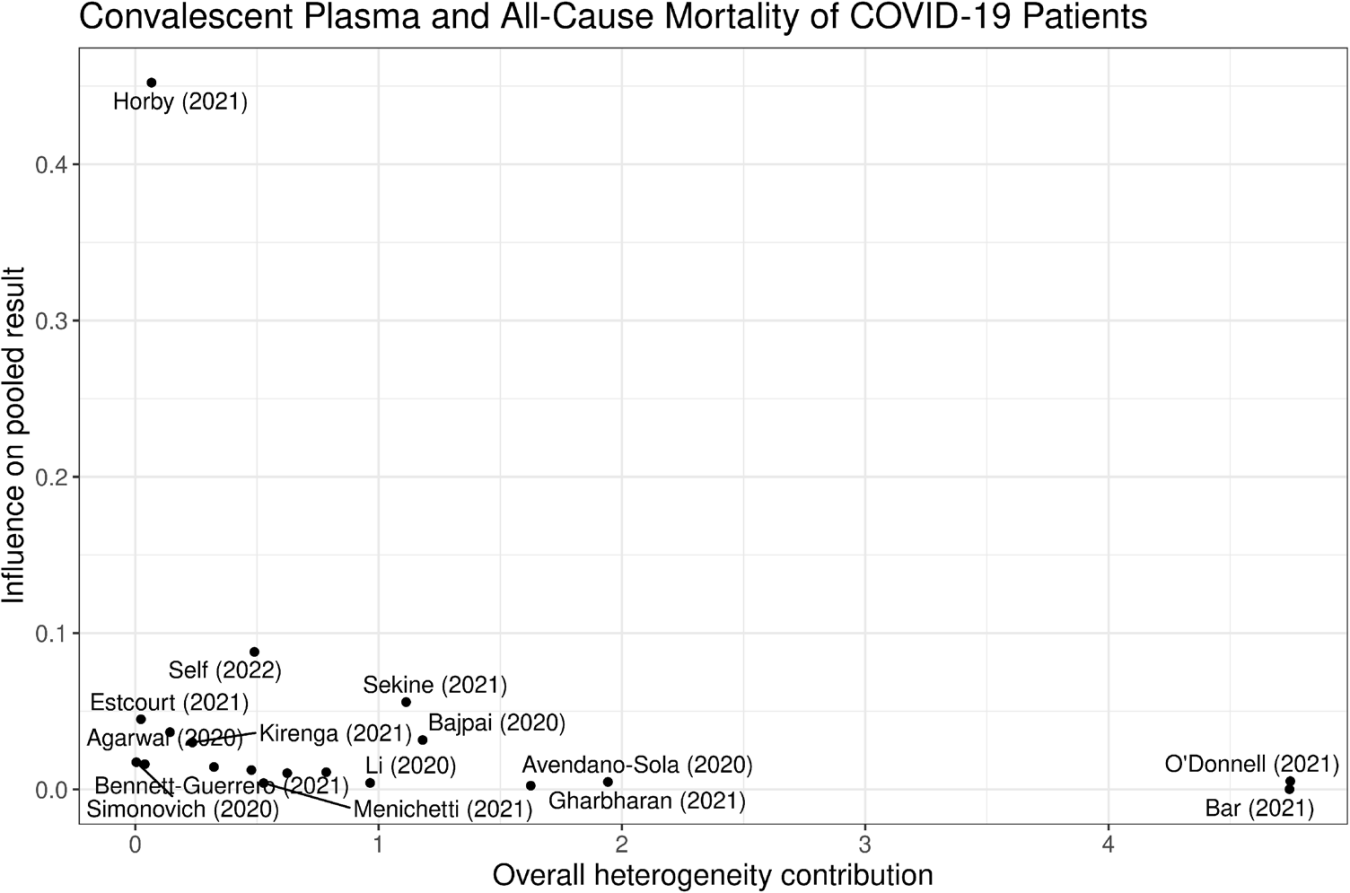
Baujat plot. Baujat plot of the selected studies showing the contribution of each study to the overall between-study heterogeneity (abscissa) and its influence on the magnitude of the pooled effect size (ordinate).

**Figure 5:**
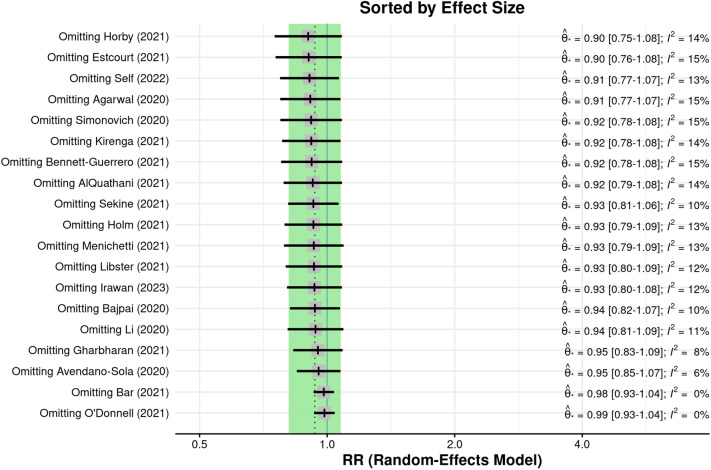
Results of a leave-one-out sensitivity analysis. Results of a leave-one-out sensitivity analysis on the pooled risk ratio effect size (Θ) estimated with a random effects model. The results are sorted by effect size and values of the I^2^ statistic are displayed. The dotted line refers to the mean estimated based on all available studies, and the green area corresponds to the corresponding 95% confidence interval.

Figure 4 illustrates the influence of each study on the between-study heterogeneity using a Baujat plot^29^. The studies by Bar 2021^21^ and O’Donnell 2021^5^ feature the largest contribution to the heterogeneity, whereas the large trial by the RECOVERY Collaborative Group^18^ displays the largest influence on the pooled result. The impact of each study on the pooled effect size is further examined within a leave-one-out analysis, presented in Fig. 5. As Fig. 4 suggests, omitting the RECOVERY Collaborative Group^18^ study results in the largest change in the estimated pooled effect size with respect to the estimate based on all available studies with an estimate of RR = 0.90 (95% CI 0.75–1.08). However, as above, the results of no statistical evidence of the benefit of convalescent plasma therapy on all-cause mortality in COVID-19 patients is robustly supported by leave-one-out analysis.

### Publication bias

**Figure 6:**
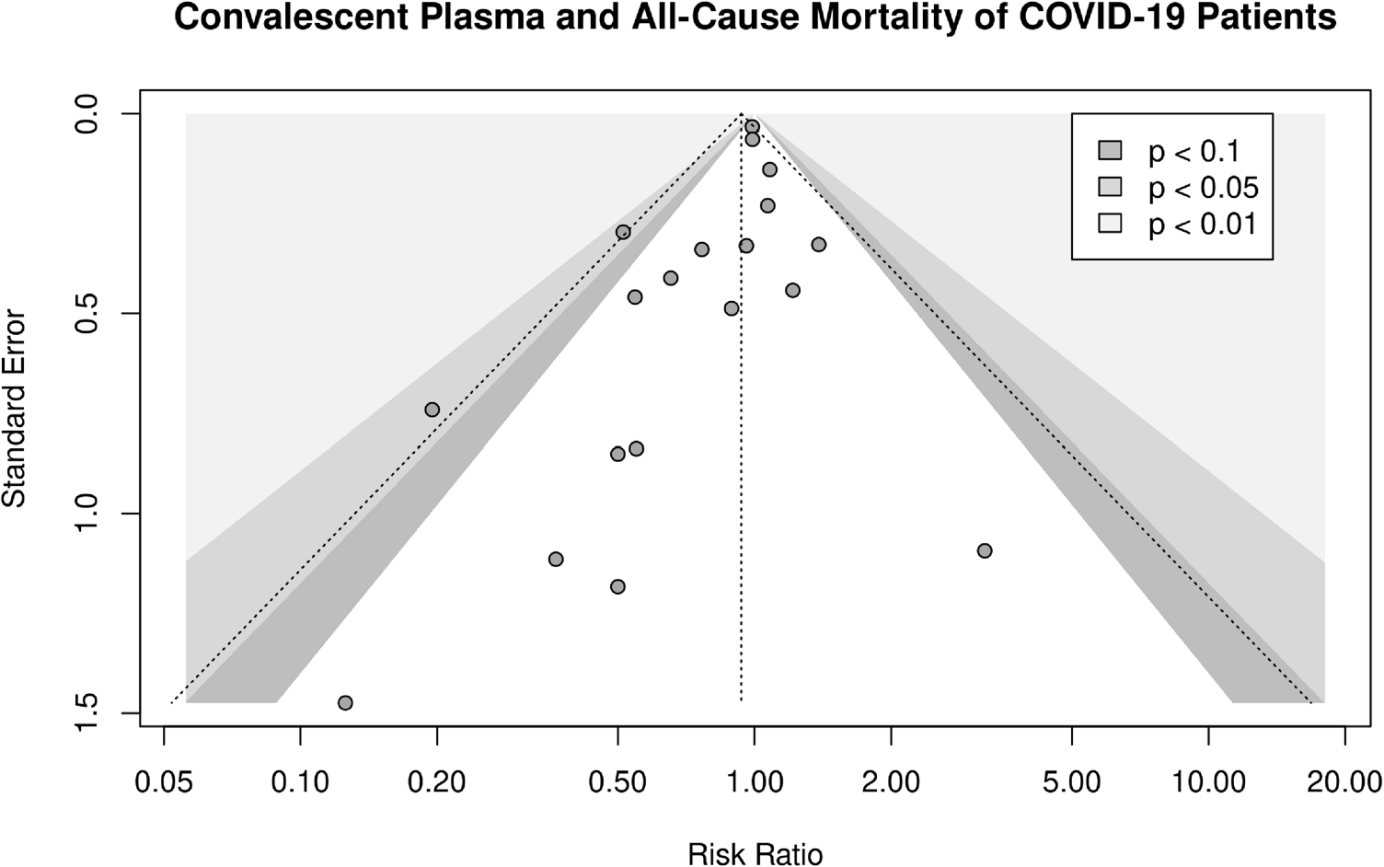
Contour-enhanced funnel plots. Contour-enhanced funnel plots showing different p-value thresholds of the studies considered in this meta-analysis. For each study, the estimated mean risk ratio was plotted against its estimated standard error.

We conclude by examining the small-study effects in the context of publication bias, shown with a Funnel Plot in Fig. 6. Visual inspection suggests no significant asymmetry, which is supported by the result of a Peters’ regression test of asymmetry (p = 0.50). Thus, no significant small-study effects could be detected in the set of studies considered here.

### Meta-regression

The median time to transfusion was available in 14 of the 19 studies considered, and varied between 3 days (minimum; study by Libster^7^) to 30 days (maximum; study by Li^6^). Time to transfusion was not associated with the studies’ effect size (p = 0.50).

## Discussion

In this meta-analysis we focused exclusively on the impact of convalescent plasma treatment on all-cause mortality in COVID-19 patients. This was different from other meta-analysis^30^, which investigated other aspects of clinical benefit, such as ICU admission rate or need for mechanical ventilation in patients who received convalescent plasma therapy. Our investigation is unique in study selection, because we included only high-quality RCTs with a control group, and we excluded all other types of study-design in order to increase the homogeneity of the collected data.

As a main result we observed no statistically significant difference in all-cause mortality between the group of patients who received convalescent plasma treatment with the best standard medical care, and the group of patients who received the best standard medical care alone or with placebo. Some of the included RCTs^4,5,7,21^ reported a statistically significant reduction in all-cause mortality after the transfusion of convalescent plasma. However, in the study by Avendano-Sola the clinical course had been followed for 15 days, in contrast with other RCTs, in which there had been a longer follow-up period of between 28 and 30 days^13,15,20^. In another included study a significantly lower all-cause mortality rate was observed in a 28-day follow-up period after convalescent plasma therapy. However, this was not associated with other clinical benefits; therefore, the patients remained hospitalized at their baseline clinical status^5^. Nevertheless, Bar et al.^21^ reported a significant clinical improvement and a reduction in mortality in the group of patients who received two units of convalescent plasma on the same day compared with the standard treatment group. In this study, the authors focused on high-risk patients with multiple coexisting conditions, particularly immuno-deficiencies and cancer (27% of included patients). It is suggested that in a high-risk population, passive immunization may play an important role due to the co-existing immunodeficiency and potentially long-lasting seronegativity). Interestingly, Libster et al.^7^ reported a lower risk of disease progression to severe respiratory failure in older adults when plasma was transfused early in the clinical course. Similarly, Simonovich et al.^16^ observed a worse clinical outcome in patients younger than 65 years compared with the enrolled elderly.

According to our results, there is no clinical benefit in the context of reduction in mortality rate in patients who receive convalescent plasma therapy earlier in the clinical course, at the beginning of the symptoms. Most of the studies included in our meta-analysis reported a median time to plasma transfusion between 3^7^ and 10^5,23^ days after symptom onset, except Li et al. (30 days)^6^. Some authors^4,17,20^ suggest that the early administration of convalescent plasma leads to significant clinical improvement (respiratory rate, oxygen saturation, resolution of shortness of breath) and reduces the probability of disease progression. Based on the results of the included RCTs we suspect that convalescent plasma may be more effective in reducing disease progression when administered early in the clinical course, when patients have not achieved a sufficient titer of own neutralizing antibodies.

Interestingly, Bansal et al.^31^ published a meta-analysis reporting a positive impact of convalescent plasma therapy on the reduction of all-cause mortality in COVID-19 patients, in contrast with our results. However, in addition to RCTs, Bansal et al. also included retrospective observational studies, case series and case reports. As a result of a subgroup analysis performed for ten RCTs from the same meta-analysis, there was no statistically significant reduction in all-cause mortality rate in the convalescent plasma group compared to the control group^31^, as well as in our study, which included RCTs only. Nevertheless, there are several case reports and case series which describe a significant improvement in the clinical status of the examined patients after plasma therapy^32,33^. According to these results, we presume that convalescent plasma therapy will not lead to a statistically significant clinical benefit in all severely ill COVID-19 patients, but there may be a particular group of patients who can benefit from plasma transfusion. Therefore, this meta-analysis does not support the routine use of convalescent plasma in all COVID-19-infected patients, but suggests that highly vulnerable patients with coexisting immunodeficiency due to advanced age, cancer treatment, immune disorders of different etiologies (suspected seronegative patients) may benefit from convalescent plasma therapy. Moreover, convalescent plasma may be beneficial in cases of high-risk exposure, as post-exposure prophylaxis, especially in the mentioned vulnerable patient populations.

Our study has some limitations which could affect the results. First, we did not analyze the information regarding the administered plasma volume and the IgG antibody titer in the transfused plasma. It is possible that in some of the RCTs included in our meta-analysis the antibody titer was not high enough to lead to any clinical change. In the included RCTs, the single dose of convalescent plasma ranged from 200 to 350 ml and was administered once or twice on the same day or on consecutive days (with the exception of Simonovich et al.^16^, where a single dose of 500 ml was administered). However, we assume that the titer of neutralizing antibody in each convalescent plasma unit can be considered a determining factor for the efficacy of convalescent plasma therapy. We hypothesize that the antibody content of plasma may be a more important factor than plasma volume; however, information on antibody titer is not available in all included studies. In addition, the timing of convalescent plasma administration seems to play an important role, and some authors^5,7^ suggest that early intervention (72 h (Libster et al.^7^) or 7 days (O’Donnel et al.^5^) after symptom onset) is critical for clinical efficacy.

Second, in our meta-analysis we did not include any information regarding other medications administered to the patients in the analyzed RCTs. Thus, which medications and procedures formed the “best standard medical care” in each particular RCT was not well defined, and could lead to inaccuracy in the results. Third, there is no information on the specific variants responsible for each infection in the included studies. We can only guess which variant might be the cause of the underlying clinical condition based on the date (months) of patient inclusion. However, the study by O’Donnel et al.^5^ highlights a positive aspect of convalescent plasma therapy compared with engineered vaccines or monoclonal antibodies. Convalescent plasma is an excellent source of polyclonal antibodies and is therefore highly adaptable to rapidly changing viral variants. Individuals (potential plasma donors) are exposed to and respond to the local viral ecology, so antibody production also changes depending on the underlying viral infection. The strength of our study is that we included high-quality peer-reviewed RCTs, all of which had control group, and therefore we provide comprehensive information about the efficacy of convalescent plasma therapy in reducing mortality rate in a general population infected with COVID-19.

## Methods

This systematic review and meta-analysis was prospectively registered on August 26, 2021 with the international Prospective Register of Systematic Reviews (PROSPERO registration number: CRD42021243629). Results are reported according to the Preferred Reporting Items for Systematic Reviews and Meta-Analyses (PRISMA) statement^34^.

### Search strategy

The literature search was conducted by two authors (NM, GE) independently. The systematic bibliographic research was conducted on the September 15, 2021 by searching for full-length articles focusing on the efficacy of convalescent plasma in reducing all-cause mortality in patients with COVID-19. Major medical databases screened were PubMed, Cochrane Library, Medline and Google Scholar. The search strategy consisted of the combination of the following mesh terms: *convalescent plasma, plasma therapy, COVID-19, SARS-CoV-2, and mortality*. After the bibliographic search was performed, all the potentially eligible titles and abstracts were screened, and full-length articles potentially meeting the inclusion criteria were evaluated. A manual search of the references of the included studies was also performed in order to supplement the electronic search. Renewed bibliographic search was conducted on January 29, 2022 and June 24, 2023 using the same databases and the same mesh terms.

### Eligibility criteria

The eligibility criteria for the meta-analysis were the following: (a) randomized controlled trials (RCTs) involving hospitalized patients with COVID-19; (b) studies analyzing the use of convalescent plasma as a treatment method in patients with COVID-19 in comparison the best medical treatment or placebo (as a control group); (c) studies with information about all-cause mortality rate; (d) full-text articles and (e) English-language literature. The reviewed studies included in our analysis focused on adult patients (˃ 18 years of age). Case reports, case series, observational studies, and studies investigating pregnant women were excluded.

### Trial selection and risk of bias assessment

Titles and abstracts were independently screened by two reviewers (NM, GE). Any relevant full-text articles were further analyzed for eligibility using the pre-defined inclusion criteria. The same investigators independently performed data collection and all authors performed the analysis and interpreted the data together. Two reviewers (NM, GE) independently evaluated the risk of bias in each study using the revised Cochrane risk-of-bias tool for randomized trials^28^. This method assessed a fixed set of potential sources of bias. Areas assessed included aspects of study design, conduct, and reporting. An assessment of the risk of bias resulting from the randomization process, deviations from planned measures, missing outcome data, outcome measurement, and selection of the reported outcome, as well as the overall risk of bias, could be rated as “low” or “high” risk of bias or indicate “some concern”. Overall risk of bias was rated as the least favorable score across all five domains. Any discrepancies were resolved by discussion between the authors.

### Data collection and analysis

Once the studies were determined to meet the inclusion criteria, two reviewers (NM, GE) independently reviewed and extracted the data related to trial design, total number of participants, age and gender of patients, disease severity, all-cause mortality rate and comorbidities of the patients for each eligible study. For the patients in the intervention arm, information was collected regarding the timing of convalescent plasma treatment from symptom onset, and amount of administered plasma. Data were collected and tabulated using Microsoft Excel Version 2019 (Microsoft Corporation, Redmond, Washington, US). The extracted data were reviewed for accuracy by all authors.

### Primary outcome and effect sizes

The binary primary outcome was all-cause mortality and the risk ratio (RR) of the convalescent plasma treatment group versus best standard medical care (control group) and its associated standard error was chosen as effect size.

### Statistical model and pooling of effect sizes

A random-effects model was employed to statistically pool the effect sizes of the selected studies: the variance of distribution of true effect sizes (τ^2) and its associated 95% confidence interval were estimated by the Paule-Mandel (PM)^35^ procedure. A Knapp-Hartung adjustment^36^ was used to calculate the confidence interval around the pooled effect. Sensitivity studies investigating the effect of the variance estimator replaced the PM-procedure with the Restricted Maximum Likelihood and Maximum Likelihood estimation^37^, the Empirical Bayes estimation^38^, DerSimonian-Laird estimation^39^ and the Sidik-Jonkman^40^ procedure as well as omitting the Knapp-Hartung adjustment. To pool the effect sizes of the selected studies, the Mantel–Haenszel method without continuity correction was used to calculate the weights of studies with binary outcome data^41,42^. The between-study heterogeneity was quantified with the I^2^ statistic and its associated 95% confidence intervals^43^ and prediction intervals based on the *t-*distribution are shown^44^.

### Influence analysis

We examined the influence of each selected study on the pooled effect sizes with two methods: a diagnostic Baujat plot^29^ was used to examine the contribution of each study to the between-study heterogeneity. We further examined the influence of each study within a leave-one-out framework and presented pooled effect sizes and I^2^ values when each study is systematically removed from the entire set of selected studies.

### Publication bias assessment

To investigate the small-study effects in the context of possible publication bias, we visually inspected contour-enhanced funnel plots^45^ for asymmetry and formally examined possible asymmetry with Peters’ regression test^46^, in which the effect size is log-transformed first and subsequently regressed on the inverse of the sample size.

### Meta-regression

For exploratory analysis, we used a mixed-effect model accounting both for sampling error and between-study heterogeneity to assess the linear relationship between effect size and the study-level predictor “median time to plasma transfusion” (in days) in a meta-regression framework. Model fit was examined by the percentage of between-study heterogeneity attributable to the predictor. Statistical significance of the predictor and its associated p-value were assessed with a t-distributed test statistic using the Knapp-Hartung adjustment.

### Statistical significance and statistical software

A p-value < 0.05 was considered statistically significant. All computations were performed with R version 4.0.5^47^, in particular with the packages meta^48^, metafor^49^ and dmetar^50^.

### Supplementary Information

Supplementary Information 1.

Supplementary Information 2.

Supplementary Information 3.

## Data Availability

All data can be provided upon request to the corresponding author.
