# Supplementary material for: Convalescent plasma and all-cause mortality of COVID-19 patients: systematic review and meta-analysis
Source: Sci Rep. 2023 Aug 9;13:12904. doi: 10.1038/s41598-023-40009-8 (PMC10412555; doi:10.1038/s41598-023-40009-8)
Supplement: Supplementary file 1 — Supplementary Information 1. [file 41598_2023_40009_MOESM1_ESM.pdf]

Estimators of the Between-Study Heterogeneity	Hartung-Knapp Adjustment	Risk Ratio (RR)	τ^2	p-value
Paule-Mandel	Yes	0.94 (0.81 - 1.08)	0.01 (0.00 - 0.35)	0.333
DerSimonian-Laird	Yes	0.96 (0.87 - 1.07)	0.00 (0.00 - 0.35)	0.456
Restricted Maximum Likelihood	Yes	0.98 (0.92 - 1.04)	0.00 (0.00 - 0.35)	0.494
Maximum Likelihood	Yes	0.98 (0.92 - 1.04)	0.00 (0.00 - 0.35)	0.494
Sidik-Jonkman	Yes	0.83 (0.66 - 1.04)	0.19 (0.00 - 0.35)	0.106
Empirical Bayes	Yes	0.94 (0.81 - 1.08)	0.01 (0.00 - 0.35)	0.333
Paule-Mandel	No	0.94 (0.82 - 1.07)	0.01 (0.00 - 0.35)	0.320
DerSimonian-Laird	No	0.96 (0.88 - 1.05)	0.00 (0.00 - 0.35)	0.429
Restricted Maximum Likelihood	No	0.98 (0.93 - 1.03)	0.00 (0.00 - 0.35)	0.461
Maximum Likelihood	No	0.98 (0.93 - 1.03)	0.00 (0.00 - 0.35)	0.461
Sidik-Jonkman	No	0.83 (0.63 - 1.10)	0.19 (0.00 - 0.35)	0.196
Empirical Bayes	No	0.94 (0.82 - 1.07)	0.01 (0.00 - 0.35)	0.320

Supplement 1.

Sensitivity analysis of the estimated pooled risk ratio (RR) and the estimated variance of the distribution of true effect sizes (τ2) with regard to the choice of the estimators of the between-study heterogeneity and the choice of Hartung-Knapp adjustment. Mean and 95% confidence intervals are shown for RR and τ2, and the p-values for the pooled effect size are shown.
