# Supplementary material for: Convalescent plasma and all-cause mortality of COVID-19 patients: systematic review and meta-analysis
Source: Sci Rep. 2023 Aug 9;13:12904. doi: 10.1038/s41598-023-40009-8 (PMC10412555; doi:10.1038/s41598-023-40009-8)
Supplement: Supplementary file 2 — Supplementary Information 2. [file 41598_2023_40009_MOESM2_ESM.pdf]

Supplement 2.
Summary of all study data.

Author	Year	Study type	Overall number	Number_Plasma	Number_Controll
**Li**	2020	RCT	103,00	52,00	51,00
**Agarwal (PLACID trial)**	2020	RCT	464,00	235,00	229,00
**Horby (RECOVERY trial)**	2021	RCT	11558,00	5795,00	5763,00
**Simonovich**	2020	RCT	333,00	228,00	105,00
**Libster**	2021	RCT	160,00	80,00	80,00
**Gharbharan**	2021	RCT	86,00	43,00	43,00
**AlQuathani**	2021	RCT	40	20	20
**Bennett-Guerrero**	2021	RCT	74	59	15
**O'Donnell**	2021	RCT	223	150	73
**Avendano-Sola**	2020	RCT	81	38	43
**Bajpai**	2020	RCT	29	14	15
**Kirenga**	2021	RCT	136	69	67

**Bar**	2021	RCT	79	40	39
**Menichetti**	2021	RCT	473	232	241
**Sekine**	2021	RCT	160	80	80
**Holm**	2021	RCT	31	17	14
Estcourt (REMAP-CAP trial)	2021	RCT	1987	1078	909
Self	2022	RCT	960	487	473
Irawan	2023	RCT	44	21	23

Age_median/mean_P	Age_range/SD_P	Age_median/mean_C	Age_range/SD_C	Male_P	Male_P_percent
70,00	62-80	69,00	63-76	27,00	51,90
52,00	42-60	52,00	41-60	177,00	75,00
63,50	14,70	63,40	14,60	3643,00	63,00
62,50	53-72.5	62,00	49-71	161,00	70,60
76,40	8,70	77,90	8,40	26,00	32,00
61,00	56-70	63,00	55-77	29,00	67,00
52,6	14,9	50,7	12,5	17,00	85,00
67	15,8	64	17,4	36,00	61,00
60	48-71	63	49-72	96,00	64,00
59		59			54,30
48,1	9,1	48,3	10,8	11	78,6
48	35-64	53	44-61	48	69,6

63	52-74			21	52,5
65	55-74	63	54-74	150	64,7
59	48-68,5	62	49,5-68	49	61,2
80	60-86	65	43-84	11	65
61	52-69	61	52-70	727	67,4
60	50-70	60	49-70	281	57,7
57	53-69	56	42-59,5	15	71,4

Male_C	Male_C_percent	Female_P	Female_P_percent	Female_C	Female_C_percent	Dis. severity
33,00	64,70	25,00	48,10	18,00	35,30	3,00
177,00	77,00	58,00	25,00	52,00	23,00	2,00
3787,00	66,00	2152,00	37,00	1976,00	34,00	3,00
64,00	61,00	67,00	29,40	41,00	39,00	3,00
34,00	42,00	54,00	68,00	46,00	58,00	1,00
33,00	77,00	14,00	33,00	10,00	23,00	3,00
15	75	3,00	15,00	5,00	25,00	3,00
8	53,3	23,00	39,00	7,00	46,70	3,00
51	70	54,00	36,00	22,00	30,00	3,00
	54,3					3,00
11	73,3	3	21,4	4	26,7	3
49	73,1	21	30,4	18	26,9	2

15	38,5	19	47,5	24	61,5	3
154	63,9	82	35,3	87	36,1	3
44	55	31	38,8	36	45	4
8	57	6	35	6	43	3
618	68	351	32,6	291	32	4
269	56,9	206	42,3	204	43,1	3
13	56,5	6	28,6	10	43,5	3

Follow up (days)	Time to transf._median (days)	Time to transf._range/SD	Mortality_P	Mortality_P_percent	Mortality_C
28,00	30,00	20-39	8,00	15,70	12,00
28,00	8,00	6-11	34,00	15,00	31,00
28,00	9,00	6-12	1399,00	24,00	1408,00
30,00	8,00	5-10	25,00	11,00	12,00
28,00	3,00		2,00	2,00	4,00
15,00	9,00	7-13	6,00	14,00	11,00
28,00			1,00	5,00	2,00
28,00	9,00	6-15	14,00	24,00	4,00
28,00	10,00	7-13	19,00	12,60	18,00
15,00	8,00		0,00	0,00	4,00
28			3	21,4	1
28	7	4-8	10	14,5	8

28	6	4-8,5	2	5	10
30	7	5-9	14	6,06	19
28	10	8-12	18	22,5	13
28			2	12	3
28			352	33	300
28	8	5-10	89	18,5	80
28			1	4,8	3

Mortality_C_percent	CRP_P_median	CRP_P_range/SD	CRP_C_median	CRP_C_range/SD	DM_P
24,00	20,40	17,00	8,87	14642,00	9,00
14,00	41,60	14-90	41,70	12-126	113,00
24,00					1535,00
11,40					40,00
5,00					23,00
26,00	84,00	50-133	109,00	70-165	13,00
10,00	110,00	63,00	91,00	52,00	7,00
27,00	3,10	0,9-9,3	1,90	0,7-8,1	19,00
24,60					55,00
9,30					

6,7

11,9

17

25,6					13
7,94					46
16,3	117,4	60,9-203,2	90,6	56,8-155,3	34
21	72	40-120	100	58-133	
33					339
17,2					176
13	460	315-965	540	337,5-1360	3

DM_P_percent	DM_C	DM_C_percent	HTA_P	HTA_P_percent	HTA_C	HTA_C_percent
17,30	12,00	23,50	29,00	55,80	27,00	52,90
48,00	87,00	38,00	92,00	39,00	81,00	35,00
26,00	1569,00	27,00				
17,50	21,00	20,00	111,00	48,70	48,00	45,70
29,00	13,00	16,00	62,00	78,00	52,00	65,00
30,00	8,00	19,00	11,00	26,00	11,00	26,00
35,00	9,00	45,00	5,00	25,00	5,00	25,00
32,20	6,00	40,00	40,00	67,80	11,00	73,30
37,00	27,00	37,00	53,00	35,00	22,00	30,00
24,6	15	22,4	24	34,8	25	37,3

32,5	19	48,7	23	57,5	30	76,9
19,8	45	18,7	82	35,3	97	40,3
42,5	29	36,3	49	61,3	49	61,3
			7	41	6	42
31,4	268	29,5				
36,4	150	31,8	303	63,8	269	57,2
14,3	8	34,8	8	38,1	10	43,5

Renal failure_P	Renal f_P_percent	Renal failure_C	Renal f_C_percent	Lung disease_P	Lung disease_P_percent	Lung disease_C
2,00	3,90	4,00	7,80			
8,00	3,00	9,00	4,00	8,00	3,00	7,00
323,00	6,00	293,00	5,00	1385,00	24,00	1328,00
10,00	4,40	4,00	3,80	32,00	14,00	7,00
1,00	1,00	3,00	4,00	3,00	4,00	3,00
1,00	2,00	6,00	14,00	12,00	28,00	11,00
1,00	5,00	1,00	5,00	3,00	15,00	0,00
7,00	11,90	0,00	0,00	7,00	11,90	2,00
13,00	9,00	8,00	11,00	15,00	10,00	5,00
				5	7,3	0

11	27,5	15	38,5	11	27,5	12
6	2,6	16	6,6	13	5,6	14
				13	16,3	9
90	18,5	80	16,9	125	25,7	135
				1	4,8	0

Lung disease_C_percent	Cancer_P	Cancer_P_percent	Cancer_C	Cancer_C_percent	Mechanical vent._P
	3,00	5,80	0,00	0,00	14,00
3,00	1,00	0,40	0,00	0,00	19,00
23,00					302,00
6,70	27,00	11,90	14,00	13,40	19,00
4,00	4,00	5,00	2,00	2,00	2,00
26,00	5,00	12,00	3,00	7,00	5,00
0,00					4,00
13,30					11,00
7,00					17,00

3

0

30,8	10	25	11	28,2	5
5,8	10	4,3	7	2,9	
11,3					34
					0
28,5	34	7	44	9,3	
0	1	4,8	2	8,7	

Mech.vent._P_percent	Mech.vent_C	Mech.vent_C_percent
27,50	11,00	22,00
8,00	19,00	8,00
5,00	315,00	5,00
8,30	10,00	9,50
2,50	4,00	5,00
12,00	8,00	19,00
20,00	6,00	30,00
18,60	3,00	20,00
11,00	11,00	15,00
21,4	1	6,7

12,8	10	25,6
42,5	34	42,5
0	1	7
